# Dual-graph attention autoencoder for spatial domain identification in ischemic stroke

**DOI:** 10.3389/fnins.2026.1881087

**Published:** 2026-07-31

**Authors:** Yuan-Yuan Chen, Wang-Ting Hu, Gang Zhang, Xian-Liang Rao, Tao Jiang

**Affiliations:** 1 Department of Neurosurgery, The First Affiliated Hospital of Anhui Medical University, Anhui Public Health Clinical Center, Hefei, Anhui, China; 2 Department of Rehabilitation Medicine, The First Affiliated Hospital of Anhui Medical University, Anhui Public Health Clinical Center, Hefei, Anhui, China

**Keywords:** attention fusion, dual-graph autoencoder, graph attention network, ischemic stroke, spatial domain identification, spatial transcriptomics, tissue disorganization

## Abstract

**Introduction:**

Spatial transcriptomics enables molecular mapping of ischemic stroke tissue, but spatial domain identification is challenging when injury disrupts normal tissue geometry. Methods relying on a single spatial-proximity graph cannot connect physically distant spots that share damage-associated transcriptional programs.

**Methods:**

We developed SpatialDomainAE, an unsupervised dual-graph attention autoencoder that constructs separate spatial-neighbor and expression-similarity graphs, processes each using graph attention, and combines their embeddings through learned per-spot fusion weights. The method was evaluated on a mouse middle cerebral artery occlusion 10× Visium dataset comprising control, 1-, 3-, and 7-day post-injury sections, totaling 10,173 spots and 22 annotated domains. All methods were evaluated over 10 random seeds using a shared Leiden-resolution protocol.

**Results:**

At 3 days post-injury, SpatialDomainAE achieved an ARI of 0.700 ± 0.025 and significantly exceeded all external baselines, including the dual-view Spatial-MGCN. Across all four samples, its performance was competitive rather than uniformly superior, and it was robust under a fixed clustering resolution. No comparable advantage was observed on an external human dorsolateral prefrontal cortex benchmark. Controlled experiments showed that the long-range transcriptomic content of the feature graph, rather than edge length alone, accounted for the improvement. Fusion weights separated lesion-associated domains at the region level, while differential expression and pathway enrichment recovered inflammatory, complement, gliosis, and proliferative programs.

**Discussion:**

SpatialDomainAE is particularly useful in disrupted pathological tissue. Its fusion weights should be interpreted as an exploratory region-level model diagnostic rather than a validated spot-level biomarker.

## 1 Introduction

Ischemic stroke affects approximately 12.2 million people per year and remains a leading cause of death and long-term disability (Virani et al., 2021; Campbell and Khatri, 2020). The ischemic lesion is organized into spatially distinct zones: a core, where neurons undergo irreversible death, and a surrounding penumbra, where tissue is still salvageable; these zones evolve over the first days after onset (Dirnagl et al., 1999; Iadecola and Anrather, 2011). Superimposed on this injury gradient, immune cell infiltration and glial activation create additional spatial structure that varies with distance from the lesion boundary (Shi et al., 2019). Mapping these zones at transcriptomic resolution is a prerequisite for identifying spatially restricted therapeutic targets.

10x Visium spatial transcriptomics (Ståhl et al., 2016) measures genome-wide expression across thousands of spots while retaining their coordinates within the tissue section (Moses and Pachter, 2022). The technology is a natural fit for stroke, where the biology is inseparable from spatial organization. Zucha et al. (2024) recently published a Visium time course from a mouse middle cerebral artery occlusion (MCAO) model, control, 1, 3, and 7 days post-injury (DPI), with 22 annotated spatial domains covering both normal brain anatomy and ischemic damage subtypes.

A central computational problem in spatial transcriptomics is spatial domain identification: assigning each spot to a tissue region on the basis of its expression and location. Graph neural network methods dominate this task. The most widely used methods build a single spatial proximity graph: SpaGCN (Hu et al., 2021) integrates expression, coordinates, and histology through graph convolutional networks; STAGATE (Dong and Zhang, 2022) trains a graph attention autoencoder; and GraphST (Long et al., 2023) adds a graph contrastive objective. Multi-modal extensions then incorporate histology or multi-omics through richer fusion modules (Long et al., 2024; Xiao et al., 2025; Huang et al., 2025).

Beyond the single-graph setting, a growing family of methods constructs two or more views, typically a spatial-proximity graph together with an expression- or pathway-similarity graph, and combines them with multi-view graph encoders or attention. Spatial-MGCN (Wang et al., 2023) and the contrastive multi-view DMGCN (Liang et al., 2025) learn view-specific and shared embeddings from a spatial graph and an expression graph and reconstruct counts with a zero-inflated negative binomial (ZINB) decoder; Path-MGCN (Zhou et al., 2025) adds a pathway-activity view, and M-STGCN (Chen et al., 2026) adds a position-aware multimodal (histology) view that jointly identifies domains and denoises expression; GAAEST (Wang et al., 2024) couples a graph-attention autoencoder with multi-level contrastive learning; and SpaMask (Min et al., 2025) and GATES (Xiao et al., 2024) use masked or globally-fused graph attention. We therefore do not claim the dual-graph construction, the per-spot attention fusion of views, or the use of graph attention as novel in themselves: building separate spatial and feature graphs, combining them with per-spot attention, and processing graphs with attention are all established designs [our fusion module itself follows SpatialGlue (Long et al., 2024), and several multi-view methods above use a comparable per-node view-attention or attention-based encoders]. Our contribution is instead analytical rather than architectural: a focused analysis of *when* and *why* the feature graph helps, an interpretability analysis of the learned fusion weights, and a targeted study of disrupted (ischemic stroke) tissue, using a deliberately simple graph-attention dual-graph autoencoder, as detailed below.

Two issues are especially relevant in pathological tissue. First, a spatial proximity graph cannot link distant spots that share a transcriptomic damage program. This matters in stroke, where related injury signatures can appear on opposite sides of the lesion or in disconnected penumbral fragments. Second, methods that aggregate each graph with fixed graph-convolution coefficients use weights that are determined by node degree rather than by content, and so cannot adapt to local context; at a domain boundary, such as the edge of the ischemic core, this mixes signal across adjacent domains, whereas learned per-edge attention can down-weight neighbors that belong to a different domain.

SpatialDomainAE is designed around this distinction. We construct two *k*-nearest neighbor graphs per spot, one from spatial coordinates and one from expression correlation, and process each with a single-layer multi-head graph attention network (GAT) (Veličković et al., 2018), using one layer per graph to limit over-smoothing (Li et al., 2018). In contrast to the GCN-based multi-view methods (Spatial-MGCN, DMGCN, Path-MGCN, and M-STGCN), which aggregate neighbors within each graph with fixed graph-convolution coefficients, both of our views are processed by GAT, so neighbor weighting is learned per edge and boundary spots can down-weight neighbors from adjacent domains. The two views are then combined by a per-spot attention fusion module adapted from SpatialGlue (Long et al., 2024); this per-spot view fusion is shared with several multi-view methods rather than unique to our model. The fused embedding is compressed through a bottleneck and decoded to reconstruct gene expression, and the bottleneck embedding is clustered with the Leiden algorithm (Traag et al., 2019).

The per-spot fusion weights (α_spatial_, α_feature_) are also available as an interpretable output. A higher feature-graph weight indicates that spatial neighbors alone are less informative at that location; we examine, as an exploratory analysis, whether this signal separates lesion-associated domains from intact tissue. An annotation-independent control (Section 3.4) shows that the weights do not track a fine-scale disorganization measure, so α_feature_ is best interpreted as a region-level model diagnostic rather than a validated spot-resolved biomarker. To test the contribution of the second graph directly rather than assume it, we further probe it with controlled feature-graph experiments (pruning long-range edges, distance-matched random controls, and local-only feature edges).

In summary, our contributions are: (i) controlled experiments demonstrating that, among feature-graph variants, the long-range *transcriptomic* content of the feature graph, not merely its long edges, accounts for its accuracy at the most disrupted time point; (ii) a focused, multi-seed demonstration, with an external benchmark and a deployment-realistic (label-free) resolution analysis, that the dual-graph advantage is concentrated where tissue architecture is most disrupted (3 DPI) and that the method is robust to a fixed, untuned clustering resolution; and (iii) as an exploratory analysis, an interpretation of the per-spot fusion weights as a region-level diagnostic that separates lesion-associated domains, with an annotation-independent control showing it does *not* track a fine-scale disorganization measure. We benchmark SpatialDomainAE against external graph-based methods (STAGATE, GraphST, SpaGCN, and the dual-view Spatial-MGCN and SpaMask), conduct systematic ablation and graph-construction experiments, and validate the identified domains through differential expression and pathway enrichment analysis.

## 2 Materials and methods

### 2.1 Dataset

We used the publicly available spatial transcriptomics dataset GSE233815, generated by Zucha et al. (2024) from a mouse MCAO ischemic stroke model. The dataset comprises four 10x Visium samples: a control sample (Ctrl) and three post-injury samples collected at 1, 3, and 7 days post-injury (1DPI, 3DPI, and 7DPI). The total number of spots across all four samples is 10,173, with individual samples containing 2,417 (Ctrl), 2,587 (1DPI), 2,573 (3DPI), and 2,596 (7DPI) spots, respectively.

Each spot was annotated into one of 22 spatial domains, including normal brain anatomical regions, amygdala (AMY), caudate putamen (CP), corpus striatum (CS), cortex layers 1–4 (CTX1-4), cortex layer 5 (CTX5), cortex layer 6 (CTX6), fiber tracts (FT), glial scar (GLS), hippocampus (HIP), hypothalamus (HY), lateral ventricle (LV), pallidum (PAL), piriform area (PIR), and thalamus (TH), as well as ischemic stroke damage (ISD) subtypes defined by time point and zone: ISD1c/ISD1p (core/penumbra at 1DPI), ISD3c/ISD3p (3DPI), ISD7c/ISD7p (7DPI), and lesioned cortex layers (lCTX4-5, lCTX6). Notably, the ISD domains are time-point-specific: ISD1c/ISD1p appear only in the 1DPI sample, ISD3c/ISD3p only in 3DPI, and ISD7c/ISD7p only in 7DPI.

### 2.2 Data pre-processing

Raw gene expression counts were processed using Scanpy (Wolf et al., 2018). Let $\text{X}\in {\mathbb{Z}}_{\geq 0}^{N\times G}$ denote the raw count matrix, where *N* = 10, 173 is the total number of spots and *G* is the number of genes. We first applied library size normalization followed by log-transformation:


$$\begin{array}{l} x_{ij}^{\prime }=\log\left(1+\frac{x_{ij}}{\sum_{j^{\prime }=1}^{G}x_{ij^{\prime }}}\times 10^{4}\right) \end{array}$$ (1)

where *x*_*ij*_ is the raw count of gene *j* in spot *i*. We then selected the top 3,000 highly variable genes (HVGs) based on the normalized dispersion criterion. For each gene *j*, the mean μ_*j*_ and variance $\sigma_{j}^{2}$ are computed across all spots; genes are binned by mean expression, and within each bin the variance is normalized:


$$\begin{array}{l} d_{j}=\frac{\sigma_{j}^{2}/\mu_{j}-{\bar{\left(\sigma^{2}/\mu \right)}}_{\text{bin}\left(j\right)}}{s_{\left(\sigma^{2}/\mu \right),\text{bin}\left(j\right)}} \end{array}$$ (2)

where ${\bar{\left(\sigma^{2}/\mu \right)}}_{\text{bin}\left(j\right)}$ and $s_{\left(\sigma^{2}/\mu \right),\text{bin}\left(j\right)}$ are the mean and standard deviation of the coefficient of variation within the expression bin of gene *j*. The top 3,000 genes by normalized dispersion *d*_*j*_ were retained, yielding the input matrix **X**′ ∈ ℝ^*N*×*P*^ with *P* = 3, 000.

### 2.3 Graph construction

Graphs were constructed independently for each sample to keep the encoder, training, and embedding spaces fully per-sample (see Section 2.5). For a sample with *N*_*s*_ spots, two *k*-nearest neighbor (KNN) graphs were built, $G_{s}=\left(V,E_{s}\right)$ and $G_{f}=\left(V,E_{f}\right)$, sharing the same node set $V=\left\{1,\ldots ,N_{s}\right\}$.

#### 2.3.1 Spatial graph

For each spot *i* with spatial coordinates ${\text{c}}_{i}=\left(x_{i},y_{i}\right)\in {\mathbb{R}}^{2}$, the *k*_*s*_ = 15 nearest neighbors were identified by Euclidean distance:


$$\begin{array}{l} E_{s}=\left\{\left(i,j\right)|j\in {\text{KNN}}_{k_{s}}\left(i,∥{\text{c}}_{i}-{\text{c}}_{j}{∥}_{2}\right)\right\} \end{array}$$ (3)

#### 2.3.2 Feature graph

For each spot *i*, the *k*_*f*_ = 20 nearest neighbors were identified by correlation distance in the *P*-dimensional HVG expression space:


$$\begin{array}{l} E_{f}=\left\{\left(i,j\right)|j\in {\text{KNN}}_{k_{f}}\left(i,1-r_{ij}\right)\right\} \end{array}$$ (4)

where *r*_*ij*_ is the Pearson correlation between expression profiles of spots *i* and *j*. Average node degrees match the chosen *k* values (${\bar{d}}_{s}=15$, ${\bar{d}}_{f}=20$). Across the four samples, the spatial and feature graphs together contain 152, 595 and 203, 460 directed edges respectively. Both graphs were stored as edge index tensors for efficient message passing in PyTorch (Paszke et al., 2019).

### 2.4 Model architecture

**Figure 1:**
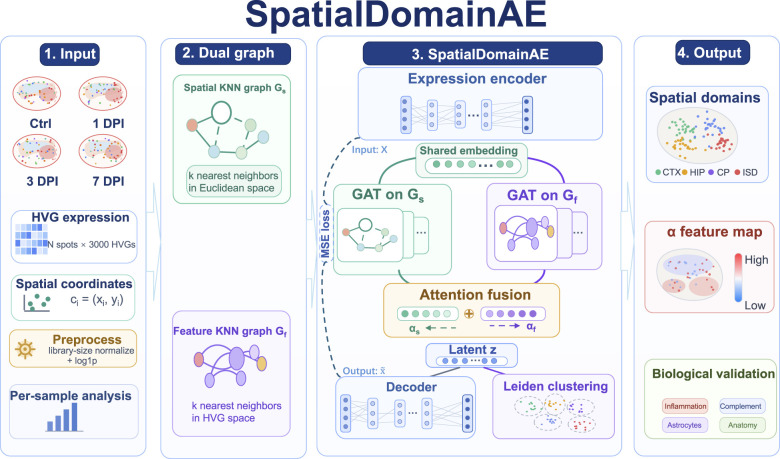
Overview of the SpatialDomainAE architecture. For each sample, an expression encoder maps highly variable gene profiles to a hidden representation; two single-layer graph attention networks process this representation on a spatial KNN graph and a feature (correlation) KNN graph; a per-spot attention fusion module produces weights (α_spatial_, α_feature_) that combine the two graph embeddings; a linear bottleneck and MLP decoder reconstruct the expression input. The fused embedding is clustered with Leiden to assign domains, and the per-spot fusion weights serve as a model-derived, region-level diagnostic of lesion-associated domains (Section 3.4).

The SpatialDomainAE architecture has four components: an expression encoder, a dual-graph GAT, an attention fusion module, and a latent bottleneck with decoder (Figure 1). Details that affect the ablation comparisons are specified below.

#### 2.4.1 Expression encoder

A three-layer MLP maps the *P*-dimensional expression vector ${\text{x}}_{i}\in {\mathbb{R}}^{P}$ to a *d*-dimensional hidden representation ${\text{h}}_{i}^{\left(\text{local}\right)}\in {\mathbb{R}}^{d}$ (with *d* = 256). Each intermediate layer applies a linear transformation, LeakyReLU activation (negative slope 0.2), Layer Normalization (Ba et al., 2016), and dropout (*p* = 0.3); the hidden widths are *P* → 512 → 256 → 256. Writing LN for Layer Normalization and ϕ(·) = LeakyReLU(·, 0.2),


$$\begin{array}{l} {\text{h}}_{i}^{\left(\text{local}\right)}=\text{Enc}\left({\text{x}}_{i}\right),{\text{u}}^{\left(\ell \right)}=\text{Dropout}\left(\text{LN}\left(\phi \left({\text{W}}^{\left(\ell \right)}{\text{u}}^{\left(\ell -1\right)}+{\text{b}}^{\left(\ell \right)}\right)\right)\right), \end{array}$$ (5)

with ${\text{u}}^{\left(0\right)}={\text{x}}_{i}$.

#### 2.4.2 Dual-graph GAT

Two independent single-layer multi-head GAT (Veličković et al., 2018) modules process **h**^(local)^ on the spatial and feature graphs. We give the equations for one graph $G\in \left\{G_{s},G_{f}\right\}$ with neighborhood N(i); the spatial and feature branches use independent weights. For attention head *m* with projection **W**_*m*_, the (un-normalized) coefficient of edge *j* → *i* and its softmax normalization over N(i) are


$$\begin{array}{lll} e_{ij}^{\left(m\right)} & = & \text{LeakyReLU}\left({\text{a}}_{m}^{⊤}\left[{\text{W}}_{m}{\text{h}}_{i}^{\left(\text{local}\right)}∥{\text{W}}_{m}{\text{h}}_{j}^{\left(\text{local}\right)}\right]\right), \end{array}$$

$$\begin{array}{lll} \alpha_{ij}^{\left(m\right)} & = & \frac{\exp(e_{ij}^{\left(m\right)})}{\sum_{j^{\prime }\in N\left(i\right)}\exp(e_{ij^{\prime }}^{\left(m\right)})}, \end{array}$$ (6)

where ∥ denotes concatenation and **a**_*m*_ is a learned attention vector. Each head aggregates its neighbors, the *M* = 4 heads (per-head dimension 64) are concatenated, and a residual connection, ELU non-linearity, and Layer Normalization give the per-graph embedding:


$$\begin{array}{l} g_{i}^{\left(m\right)}=\sum_{j\in N(i)}\alpha_{ij}^{\left(m\right)}W_{m}h_{j}^{\left(\text{local}\right)},\\ h_{i}^{\left(G\right)}=\text{LN​}\left(\text{ELU​}\left({\|}_{m=1}^{M}g_{i}^{\left(m\right)}+W_{\text{res}}h_{i}^{\left(\text{local}\right)}\right)\right). \end{array}$$ (7)

The learned per-edge coefficients let boundary spots down-weight neighbors from adjacent domains. A single layer per graph keeps each spot's receptive field at one *k*-hop neighborhood and avoids over-smoothing (Li et al., 2018). The two modules yield ${\text{h}}_{i}^{\left(\text{spatial}\right)}$ and ${\text{h}}_{i}^{\left(\text{feature}\right)}$.

#### 2.4.3 Attention fusion

Following Long et al. (2024), the spatial and feature embeddings are combined per spot. For each spot *i* and each view *v* ∈ {spatial, feature}, a shared non-linear projection maps the embedding to a scalar logit, and the two logits are softmax-normalized into per-spot weights:


$$\begin{array}{lll} s_{v}^{\left(i\right)}= & {\text{u}}_{\omega }^{⊤}\tanh\left({\text{W}}_{\omega }{\text{h}}_{i}^{\left(v\right)}\right),\;\alpha_{v}^{\left(i\right)}=\frac{\exp(s_{v}^{\left(i\right)})}{\sum_{v^{\prime }}\exp(s_{v^{\prime }}^{\left(i\right)})},\\ \alpha_{\text{spatial}}^{\left(i\right)} & + & \alpha_{\text{feature}}^{\left(i\right)}=1, \end{array}$$ (8)

with shared parameters ${\text{W}}_{\omega }\in {\mathbb{R}}^{d\times d}$ and ${\text{u}}_{\omega }\in {\mathbb{R}}^{d}$. The fused embedding is the weighted combination


$$\begin{array}{l} {\text{h}}_{i}^{\left(\text{fused}\right)}=\alpha_{\text{spatial}}^{\left(i\right)}{\text{h}}_{i}^{\left(\text{spatial}\right)}+\alpha_{\text{feature}}^{\left(i\right)}{\text{h}}_{i}^{\left(\text{feature}\right)}. \end{array}$$ (9)

Because the weights are learned per spot, spots in homogeneous tissue can rely on spatial context while spots in transcriptomically diverse regions can favor the feature graph; the same weights also serve as a region-level diagnostic analyzed in Section 3.4.

#### 2.4.4 Latent bottleneck and decoder

A linear projection compresses the fused embedding to a *d*_*z*_ = 64-dimensional latent vector ${\text{z}}_{i}={\text{W}}_{z}{\text{h}}_{i}^{\left(\text{fused}\right)}$, and a three-layer MLP decoder (*d*_*z*_→*d* → 512 → *P*) with LeakyReLU activations reconstructs the expression input:


$$\begin{array}{l} {\hat{\text{x}}}_{i}=\text{Dec}\left({\text{z}}_{i}\right)={\text{W}}^{\left(3\right)}\phi \left({\text{W}}^{\left(2\right)}\phi \left({\text{W}}^{\left(1\right)}{\text{z}}_{i}+{\text{b}}^{\left(1\right)}\right)+{\text{b}}^{\left(2\right)}\right)+{\text{b}}^{\left(3\right)}. \end{array}$$ (10)

The preprocessing, graph construction, and model operations are summarized in Equations 1–10. The model is trained end-to-end by minimizing the reconstruction loss of Equation 11 between ${\hat{\text{x}}}_{i}$ and the preprocessed input ${\text{x}}_{i}^{\prime }$; the reconstruction objective is computed externally on the decoder output and is not part of the decoder itself. After training, the per-sample latent embeddings {**z**_*i*_} are clustered with the Leiden algorithm (Traag et al., 2019). The full model has approximately 3.5M trainable parameters.

### 2.5 Training

The model is trained *independently for each sample* (no parameter sharing across time points) with mean squared error (MSE) reconstruction loss:


$$\begin{array}{l} L_{\text{MSE}}=\frac{1}{N_{s}}\overset{N_{s}}{\underset{i=1}{\sum }}∥{\text{x}}_{i}^{\prime }-{\hat{\text{x}}}_{i}{∥}_{2}^{2} \end{array}$$ (11)

where *N*_*s*_ is the number of spots in the sample, ${\text{x}}_{i}^{\prime }$ is the preprocessed expression vector, and ${\hat{\text{x}}}_{i}$ is the reconstruction. The MSE objective is computed externally on the decoder output, comparing the reconstruction to the input expression. Optimization uses AdamW (Loshchilov and Hutter, 2019) (learning rate 10^−3^ with cosine decay to 0, weight decay 10^−4^, global-norm gradient clipping at 1.0) for 500 epochs.

#### 2.5.1 Rationale for per-sample training

We deliberately train one model per sample rather than a single shared encoder across the four time points, for three reasons specific to this dataset. First, the injury-related (ISD) domains are mutually exclusive across time points by construction, ISD1c/ISD1p occur only at 1 DPI, ISD3c/ISD3p only at 3 DPI, and ISD7c/ISD7p only at 7 DPI (Section 2.1), so there is no shared injury-domain label space for a joint model to exploit, and the principal axis of variation across samples is the experimental time point itself. Second, a joint encoder would have to either absorb or be corrected for cross-sample (batch/time) effects; any such batch correction risks removing the very lesion-stage signal that distinguishes the ISD subtypes, conflating biological progression with a nuisance covariate. Training per sample keeps graph construction, embedding space, and clustering free of cross-sample information, so each reported score reflects that section alone. Third, the per-spot fusion weights are interpreted within a section as a relative readout of local disorganization (Section 3.4); per-sample training keeps that readout on a common within-section footing without an additional cross-sample normalization step. The trade-off is that this design does not leverage shared anatomical structure across samples and does not, by itself, test cross-sample generalization; a shared or inductive encoder (Hamilton et al., 2017) that processes new sections without retraining is a natural extension, which we return to in the Discussion.

After training, latent vectors {**z**_*i*_} are clustered with the Leiden algorithm (Traag et al., 2019) on a *k*-NN graph of the embeddings (*n*_neighbors_ = 15), maximizing modularity at resolution γ. We sweep the same fixed grid γ ∈ {0.3, 0.5, 0.8, 1.0, 1.5, 2.0, 3.0, 5.0} for *every* method (SpatialDomainAE, all ablation variants, STAGATE, GraphST, SpaGCN, and Spatial-MGCN) and report the best per-sample ARI. This is an oracle resolution-selection protocol: labels are used only after clustering to choose the reported resolution. The protocol gives a uniform upper-bound comparison across methods, but it should not be interpreted as fully unsupervised model selection in a deployment setting. To quantify run-to-run variability rather than relying on a single run, every method (our model and all baselines) was trained and evaluated across the same 10 random seeds (0–9), and the benchmark (Table 1) and all cross-method comparisons use these 10 seeds. For the internal ablation among our own variants (Supplementary Table S2), our model was additionally run twice per seed (20 runs) to increase statistical power. Unless noted otherwise, reported values are mean ± SD, and method comparisons use paired Wilcoxon signed-rank tests across the shared seeds (Section 3). We note that with *n* = 10 paired seeds the smallest attainable two-sided *p* is ≈0.002, so several strong comparisons are reported at that floor; and that we do not globally correct across all tables, so borderline results (*p*≈0.05, e.g., the dual-vs-no-graph ablation contrast) should be read as suggestive rather than definitive, whereas the *p*≈0.002 comparisons and the feature-graph contrasts (Benjamini–Hochberg-corrected; Table 2) remain significant under correction.

**Table 1:** Spatial domain identification benchmark against external graph-based baselines, including two dual-view methods (Spatial-MGCN, SpaMask).

Method	3 DPI ARI	Mean ARI	Mean NMI
SpatialDomainAE (Ours)	**0.700** **±0.025**	0.602 ± 0.016	0.737 ± 0.006
Spatial-MGCN	0.668 ± 0.027	**0.618** **±0.012**	**0.754** **±0.007**
SpaMask^†^	0.395 ± 0.030	0.359 ± 0.014	0.568 ± 0.006
STAGATE	0.563 ± 0.027	0.527 ± 0.016	0.689 ± 0.008
GraphST	0.641 ± 0.015	0.598 ± 0.007	0.743 ± 0.005
SpaGCN	0.609 ± 0.015	0.597 ± 0.012	0.748 ± 0.006

All methods use the same 10 random seeds (mean ± SD), with embeddings clustered on the shared oracle Leiden grid; the 3 DPI column is the most lesion-disrupted section and the Mean columns average over the four samples. Best per column in **bold**, second-best underlined. At 3 DPI our method significantly exceeds every external baseline (paired Wilcoxon, *p* ≤ 0.037); per-method significance and the graph-structure ablation are given in Section 3.2 and Supplementary Table S2.

^†^SpaMask uses masked autoencoding with *k*-means at a known cluster count and ranked lowest under both the oracle-Leiden protocol (shown) and its native *k*-means (mean ARI 0.359).

**Table 2:** Feature-graph content analysis (3 DPI ARI, mean ± SD over 10 seeds).

Feature-graph variant	3 DPI ARI	*p* (vs. full)
Full (correlation KNN, *ours*)	**0.714** **±0.026**	*ref*.
Prune long-range edges	0.699 ± 0.028	0.037
Local-only feature edges	0.695 ± 0.024	0.037
Distance-matched random	0.695 ± 0.022	0.004
Random (degree-matched)	0.697 ± 0.027	0.027
Spatial-only (no feature graph)	0.702 ± 0.033	0.19

Each variant replaces the feature graph; all other components are unchanged. This independent 10-seed run differs slightly in absolute value from the main benchmark (full model 0.714 vs. 0.700, within SD); the comparison of interest is the seed-paired difference of the full graph vs. each variant (*p*: paired Wilcoxon; the four edge-content contrasts survive Benjamini–Hochberg correction, adjusted *p* ≤ 0.046). The full graph significantly beats every content-degraded variant, including a distance-matched random control (equal edge length, no transcriptomic content); removing the feature graph entirely (spatial-only) gives a smaller, non-significant difference (*p* = 0.19). Bold values indicates the best-performing result.

### 2.6 Evaluation metrics

We score predicted clusterings against ground-truth domain annotations with the Adjusted Rand Index (ARI) (Hubert and Arabie, 1985) and Normalized Mutual Information (NMI) (Strehl and Ghosh, 2002). Each sample was processed and scored independently, separate graph construction, separate training, separate Leiden clustering, separate evaluation, so reported scores reflect per-sample performance and never share information across time points.

### 2.7 Benchmark methods

#### 2.7.1 Ablation variants

SpatialGATAE (spatial graph only) and ExprOnlyAE (no graph, MLP autoencoder). Together with the full SpatialDomainAE, these three variants form a no-graph, single-graph, and dual-graph series.

#### 2.7.2 External baselines

We compared against three single-spatial-graph methods, STAGATE (Dong and Zhang, 2022), GraphST (Long et al., 2023), and SpaGCN (Hu et al., 2021), and two closely related dual-view methods, Spatial-MGCN (Wang et al., 2023) and SpaMask (Min et al., 2025), which (like SpatialDomainAE) build separate spatial and expression/feature graphs. To make the comparison fair, every method received the *same* input (the per-sample 3, 000-HVG library-size-normalized, log-transformed expression matrix of Section 2.2 and the same spatial coordinates), was run over the same 10 random seeds, and had its embedding clustered with the *same* oracle Leiden resolution grid (Section 2.5); as with our model, ground-truth labels were used only after clustering, to select the reported resolution. Full method-specific implementation details (graphs, hyperparameters, epochs, random seeds, and clustering settings for each baseline) are provided in the Supplementary Methods.

#### 2.7.3 Differential expression analysis

To validate the biological coherence of identified domains, we performed Wilcoxon rank-sum tests between each cluster and all other spots. Marker enrichment was computed as the fraction of predefined marker genes present among the top 50 ranked DEGs for each cluster; adjusted *p* values and log fold changes were retained for interpretation.

## 3 Results

### 3.1 Spatial domain identification on MCAO samples

**Figure 2:**
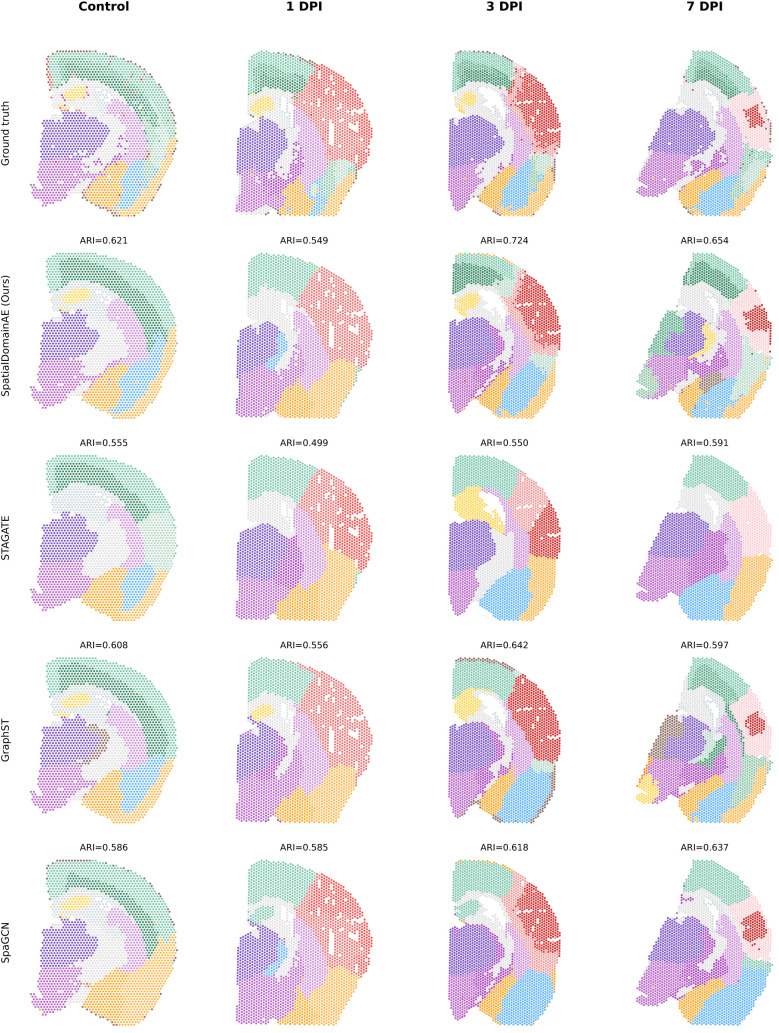
Spatial domain maps for the four samples (columns) under each method (rows): ground-truth annotations on top, then SpatialDomainAE (Ours), followed by the three external graph-based baselines. The per-sample ARI annotated on each predicted panel is for a single representative seed and is shown for visualization only; the quantitative, multi-seed comparison (mean ± SD over 10 seeds for all methods) is given in Table 1, with single-seed values lying within that run-to-run range. For each method, predicted Leiden cluster identifiers were re-labeled to match ground-truth domain colors using Hungarian assignment for visualization only; ground-truth labels are never used during training, embedding extraction, or clustering. SpatialDomainAE recovers the major anatomical boundaries and the ischemic lesion structure at every time point, with the clearest improvement over baselines visible at 3 DPI. An enlarged view of the 3 DPI lesion-bearing region [ground truth vs. ours vs. the strongest single-graph baseline (GraphST)] is shown in Supplementary Figure S1.

Figure 2 compares ground-truth annotations with the Leiden cluster maps produced by SpatialDomainAE and the three external graph-based baselines. SpatialDomainAE recovers the major anatomical boundaries (cortex layers, hippocampus, and thalamus) and delineates the ischemic lesion at every time point. The differences between methods are most visible at 3 DPI: SpatialDomainAE recovers both the ischemic core and penumbra zones with shapes that match the ground truth, whereas STAGATE under-segments the lesion (much of the ischemic territory is absorbed into adjacent anatomical clusters), and GraphST and SpaGCN split the cortex and lesion into smaller, noisier sub-regions. On the less perturbed Ctrl, 1 DPI, and 7 DPI samples, the methods produce qualitatively similar maps, consistent with their close ARI values on these sections (Section 3.2). Comparable maps for the two ablation variants (SpatialGATAE, ExprOnlyAE) are reported alongside the ablation analysis in Section 3.3.

### 3.2 Benchmark against unsupervised methods

For each sample, we trained the model independently, extracted embeddings, clustered them with Leiden using the uniform resolution grid described in Section 2.5, and scored the resulting clusters against ground-truth annotations with ARI and NMI (Table 1).

To quantify run-to-run reliability rather than relying on a single run, every method (our model and all external baselines) was trained and evaluated across the same 10 random seeds, reporting mean ± SD (Table 1); comparisons use paired Wilcoxon signed-rank tests across the shared seeds. Performance on the 3 DPI section is itself variable across seeds (range 0.660–0.758; mean 0.700 ± 0.025), so we report distributions rather than any single run.

The clearest separation occurs at 3 DPI, when both ischemic core (ISD3c) and penumbral (ISD3p) compartments are present. On this sample SpatialDomainAE obtains the highest ARI (0.700 ± 0.025) and significantly exceeds every external baseline: GraphST (0.641, Δ = +0.059, *p* = 0.002), SpaGCN (0.609, Δ = +0.091, *p* = 0.002), STAGATE (0.563, Δ = +0.137, *p* = 0.002), and the dual-view Spatial-MGCN (0.668, Δ = +0.032, *p* = 0.037). We benchmarked a second dual-view method, SpaMask, but it transferred poorly to this lesion dataset (3 DPI ARI 0.395, Δ = +0.305, *p* = 0.002; lowest of all methods under both clustering protocols), so among the dual-view baselines Spatial-MGCN is the only competitive comparator (Table 1). On Ctrl, 1 DPI, and 7 DPI the methods are closer, consistent with their similar mean ARI values.

Averaged over the four samples, SpatialDomainAE (0.602 ± 0.016) is competitive with the strongest baseline, the dual-view Spatial-MGCN (0.618 ± 0.012; difference within run-to-run noise), and exceeds STAGATE, GraphST, and SpaGCN on mean ARI; on mean NMI Spatial-MGCN leads slightly (0.754 vs. ours 0.737). We therefore do not claim a large average-accuracy advantage. Instead, the dual-graph design contributes in two specific ways. First, the per-sample picture: at 3 DPI, the section with the strongest lesion-associated heterogeneity, our method significantly exceeds every external baseline (including Spatial-MGCN, *p* = 0.037), whereas on the less perturbed sections the methods converge, exactly the behavior expected if the feature graph matters most when tissue architecture is disrupted. Second, interpretability: the per-spot fusion weights (Section 3.4) provide a region-level readout that separates lesion-associated domains and that single-graph methods do not expose. The contribution of each graph component is isolated in the ablation study (Section 3.3; full table in Supplementary Table S2), and the contribution of the long-range feature edges is tested directly with controlled experiments (Table 2).

### 3.3 Ablation study

To isolate the contribution of graph structure, we compared the full dual-graph model against two ablations sharing the same backbone and per-sample pipeline: SpatialGATAE (spatial graph only) and ExprOnlyAE (no graph). As these are internal comparisons among our own variants, they are evaluated over the full set of repeated runs (20 runs = 10 seeds × 2 repeats) to maximize statistical power. Over the four-sample mean, the dual graph significantly improves on the spatial-only variant (Δ≈+0.016 ARI, paired Wilcoxon over 10 seeds *p* = 0.010); the gain over the no-graph variant is smaller and not individually significant (Δ≈+0.011, *p* = 0.064). At 3 DPI alone the gap to the ablations is likewise small and not individually significant, indicating that much of the lesion structure is already recoverable from spatial context. The content-specific contribution of the feature graph is established more directly by the controlled experiments of Section 3.6 (Table 2), which show that destroying the transcriptomic content of the feature edges significantly degrades accuracy. Per-variant numbers are reported in Supplementary Table S2. The dual-graph design additionally produces per-spot fusion weights, an interpretability handle that neither ablation exposes.

### 3.4 Attention fusion separates lesion-associated domains

The attention fusion module produces per-spot weights (α_spatial_, α_feature_) that indicate how much each spot relies on spatial context vs. transcriptomic similarity. We treat these weights as a model-derived proxy for local tissue organization: in well-organized tissue, spatial neighbors are informative and α_spatial_ should dominate; where spatial structure is disrupted, the model may shift toward the feature graph (an expectation we test, and partly revise, with an annotation-independent control below).

**Figure 3:**
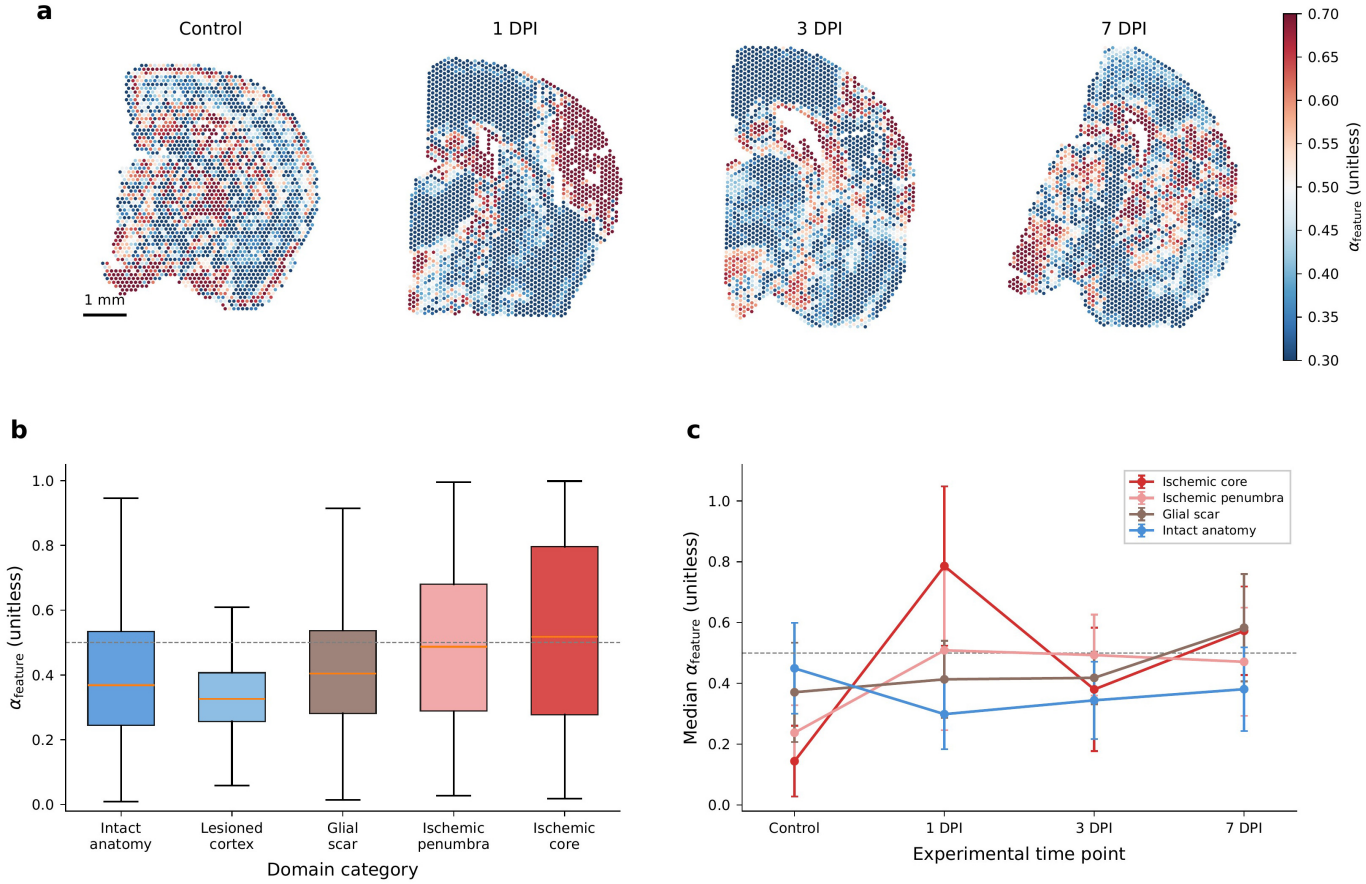
Attention fusion weights as a region-level readout that separates lesion-associated domains. **(a)** Spatial maps of α_feature_ (feature graph reliance) across the four samples. Blue: spatial-dominant (α_feature_ → 0.30); white: balanced (α_feature_ = 0.50); red: feature-dominant (α_feature_ → 0.70). At 1 DPI, the acute ischemic territory is highlighted as a contiguous red region; at 3 DPI and 7 DPI the within-lesion pattern is more heterogeneous [quantified per time point in **(c)**]. **(b)** Distribution of α_feature_ by domain category, aggregated across the four samples. Ischemic core and penumbra domains show the highest median reliance on the feature graph (~0.52 and ~0.49); anatomical regions and lesioned cortex remain spatial-dominant (~0.37 and ~0.33). Dashed line: equal weighting (α = 0.5). **(c)** Time-course of α_feature_ in lesion-related and intact domain categories. Points are per-sample medians; error bars denote IQR/2. Ischemic penumbra remains elevated relative to intact anatomy at every post-injury time point; the ischemic core shows a non-monotonic trajectory consistent with acute disorganization at 1 DPI, lesion consolidation by 3 DPI, and partial remodeling by 7 DPI.

Figure 3a maps α_feature_ across all four samples (color scale: blue α_feature_ = 0.30, white 0.50, red 0.70). The control brain shows scattered weights without strong spatial localization, reflecting intact tissue architecture in which spatial neighbors and transcriptomically similar spots largely coincide. The clearest lesion-localized pattern appears at 1 DPI, where a contiguous red region (high feature-graph reliance) overlaps the acute ischemic territory and the surrounding tissue stays predominantly blue (spatial-dominant). The 3 DPI and 7 DPI samples show more heterogeneous patterns within the lesion, which we examine quantitatively below.

Figure 3b quantifies this pattern by domain category. Ischemic core and penumbra domains show the highest median α_feature_ (~0.52 and ~0.49), at or above the equal-weighting line (α = 0.5). Glial scar regions are intermediate (~0.41), and anatomical regions remain spatial-dominant (~0.37). Lesioned cortex shows the lowest median α_feature_ (~0.33), suggesting that the laminar cortical structure still provides useful spatial context despite altered gene expression. The resulting ordering, ischemic core > ischemic penumbra > glial scar > anatomical > lesioned cortex, tracks the expected gradient of tissue disruption (Dirnagl et al., 1999; Shi et al., 2019), with the lesioned-cortex outlier reflecting that altered expression alone does not always weaken the value of spatial neighbors. The reading is a model-derived, region-level summary that separates lesion-associated domains, not a direct measurement of tissue damage.

The boxplot in Figure 3b aggregates spots across all four samples; the picture is more nuanced once we resolve it by time point. We therefore tracked α_feature_ in lesion-related categories per sample (Figure 3c). Because the per-spot weights derive from a single trained model and neighboring spots are spatially autocorrelated, we summarize these contrasts with effect sizes (Cliff's δ) rather than spot-level significance tests, which would treat autocorrelated spots as independent units and are therefore not reported. Within each post-injury sample, ischemic-region spots showed higher α_feature_ than intact-anatomy spots from the same section: at 1 DPI, ischemic core 0.786 vs. intact 0.299 (Cliff's δ = 0.63, large); at 7 DPI, 0.573 vs. 0.381 (δ = 0.38, medium); ischemic penumbra was elevated relative to intact at every time point (δ = 0.37 and 0.34, medium, at 1 and 3 DPI; δ = 0.20, small, at 7 DPI). The 3 DPI core was a notable exception: its median α_feature_ (0.380) dropped to near the intact level of the same section (0.344; δ = 0.09, negligible) and was the lowest of the three post-injury cores, consistent with a well-demarcated, internally homogeneous core in which spatial neighbors remain informative even though expression is markedly altered. We treat these per-spot patterns as an interpretability readout of a single model rather than a population-level statistical test, and anchor the readout against an annotation-independent measure in the next paragraph. Across samples, the trajectory in the core (high at 1 DPI, lowest at 3 DPI, intermediate at 7 DPI) tracks the established time course of MCAO lesions: acute disorganization at 1 DPI, lesion consolidation by 3 DPI, and partial remodeling by 7 DPI (Dirnagl et al., 1999; Iadecola and Anrather, 2011).

#### 3.4.1 Anchoring the readout to an annotation-independent measure

To test whether α_feature_ reflects more than the model's own clustering, we correlated it with a measure that does not use a spot's own domain label: the local label entropy, i.e., the Shannon entropy of ground-truth domain annotations among each spot's *k*_*s*_ spatial neighbors (high where the spatial neighborhood is heterogeneous). Across all four samples α_feature_ showed a weak *negative* Spearman correlation with local label entropy (per-sample ρ from −0.07 to −0.17; pooled ρ = −0.11, *n* = 10, 173). This indicates that α_feature_ is *not* a spot-level map of micro-scale boundary heterogeneity, if anything it is lower at mixed domain boundaries, but rather a region-level signal that is elevated within spatially-coherent yet transcriptomically-distinct lesion compartments (e.g., the internally homogeneous core/penumbra), consistent with Figure 3b. We therefore interpret α_feature_ as a model-derived diagnostic that separates lesion-associated domain categories, and explicitly not as a fine-grained, spot-resolved disorganization measurement.

### 3.5 Feature graph captures long-range transcriptomic similarity

To understand why the feature graph improves domain identification, we analyzed the physical distances spanned by edges in each graph (Supplementary Figure S2). Spatial graph edges connect nearby spots (median distance ~232–241 pixels), at the scale of the Visium center-to-center spot pitch (~100 μm). Feature graph edges span a wider range (median distance ~730–818 pixels, ~3.3 × the spatial graph median), reaching across several spot pitches and tissue compartments. These long-range connections link spots that share transcriptomic programs but are physically separated, for example, cortex layer 5 on both sides of the lesion, or disconnected fragments of the penumbra. The feature graph median distance is highest at 3 DPI (818 pixels vs. 730 pixels at 1 DPI), consistent with stronger transcriptomic heterogeneity in that section. By construction, these connections are absent from spatial-only graphs.

### 3.6 The transcriptomic content of the feature graph drives the 3 DPI gain

That feature-graph edges span long distances (Section 3.5) does not by itself show that these edges *improve* domain identification, nor that their transcriptomic content, rather than merely their length, is what helps. We therefore replaced the feature graph in SpatialDomainAE with five controlled variants and re-evaluated under the identical protocol (10 seeds; Table 2): (i) *prune long-range*, keeping only feature edges shorter than the per-sample median feature-edge distance; (ii) *local-only*, computing feature similarity but restricting each spot's feature neighbors to its 50 nearest spatial neighbors; (iii) *distance-matched random*, random edges resampled to match the physical-distance distribution of the real feature graph (so the edges are equally long-range but carry no transcriptomic information); (iv) *random*, a degree-matched uniform-random graph; and (v) *spatial-only*, removing the feature graph entirely.

At 3 DPI, the full feature graph significantly outperformed every variant that degrades edge *content* (paired Wilcoxon across seeds): pruning long-range edges reduced ARI from 0.714 to 0.699 (*p* = 0.037), restricting to local feature edges to 0.695 (*p* = 0.037), and, most informatively, the distance-matched random control to 0.695 (*p* = 0.004) and the fully random graph to 0.697 (*p* = 0.027). Because the distance-matched control preserves edge length but destroys transcriptomic content, its significant deficit isolates the contribution of the *transcriptomic similarity* carried by the long-range edges, not merely their reach. Consistent with the ablation (Supplementary Table S2), the gap to the pure spatial-only graph is smaller and not individually significant at 3 DPI (0.714 vs. 0.702, *p* = 0.19): at this consolidated time point the spatial graph already recovers much of the lesion, and the feature graph adds a small but content-specific refinement.

### 3.7 Robustness to graph-construction choices

We assessed the sensitivity of SpatialDomainAE to its construction hyperparameters by varying one choice at a time around the defaults (three seeds per setting). Performance was stable across the spatial neighborhood size *k*_*s*_∈{6, 10, 15, 20, 30} (mean ARI 0.599–0.618), the feature neighborhood size *k*_*f*_∈{10, 15, 20, 30, 50} (mean ARI 0.602–0.616), and the feature-similarity metric (Pearson correlation 0.603, cosine 0.605, Euclidean 0.600). Re-selecting highly variable genes from raw counts at {2, 000, 3, 000, 5, 000} HVGs likewise left performance stable (mean ARI 0.552–0.584, with the default 3, 000 HVGs performing best; the modest offset from the benchmark mean of 0.602 (Table 1) reflects this independent three-seed, raw-count re-selection). No setting changed the mean ARI by more than ~0.03, and the chosen defaults (*k*_*s*_ = 15, *k*_*f*_ = 20, correlation distance, 3, 000 HVGs) are within noise of the per-axis optima, indicating the method is not finely tuned to a particular construction choice.

### 3.8 Resolution selection without oracle labels

The benchmark ARIs use an oracle resolution (Section 2.5), which requires ground-truth labels and therefore reports an upper bound rather than deployable performance. To assess realistic use, we re-clustered every method's embeddings (three seeds) under two label-free criteria: a single fixed resolution (γ = 1.0 for all samples and methods) and a resolution chosen by maximizing the silhouette score (Supplementary Table S1). Under the fixed resolution, the most common deployment choice, SpatialDomainAE is the top method by a clear margin (3 DPI ARI 0.666; four-sample mean 0.585) and degrades least relative to its oracle value, whereas the dual-view Spatial-MGCN, which marginally led under the oracle, drops sharply (0.486 mean), indicating it requires per-sample resolution tuning that SpatialDomainAE does not. Under fully unsupervised silhouette-based selection the methods are closer and SpatialDomainAE is competitive but no longer best (mean 0.545; 3 DPI 0.593), with Spatial-MGCN and SpaGCN selecting better silhouette-aligned resolutions. Thus the practical advantage of SpatialDomainAE is its robustness to a fixed, untuned resolution rather than its behavior under silhouette auto-selection.

### 3.9 External validation on intact laminar cortex (DLPFC)

To test generality beyond the single MCAO dataset, we applied SpatialDomainAE and the external baselines to the human dorsolateral prefrontal cortex (DLPFC) 10x Visium benchmark (Maynard et al., 2021) (12 sections with manual cortical-layer annotations), using the identical preprocessing, graph construction, and oracle Leiden protocol (three seeds). On this dataset, whose laminar architecture is intact and well-aligned with spatial proximity, SpatialDomainAE (mean ARI 0.231 ± 0.006) performed comparably to the spatial-only ablation (0.244), STAGATE (0.223), and SpaGCN (0.258), but below GraphST (0.337), which was the strongest method here. This is the behavior anticipated by our analysis: where tissue geometry already coincides with domain identity, the long-range feature edges that benefit disrupted tissue provide no advantage and can even connect spots across adjacent layers. The DLPFC result therefore does not contradict but rather delimits our claim: SpatialDomainAE is targeted at pathological tissue with disrupted architecture (e.g., the 3 DPI ischemic lesion), where it leads all baselines, and is merely competitive on intact laminar tissue.

### 3.10 Biological validation: domain-specific gene expression

To confirm that the identified domains are biologically coherent, we performed differential expression analysis on SpatialDomainAE clusters from the 3 DPI sample (Wilcoxon rank-sum tests; 21 clusters, Leiden resolution 1.5). We chose 3 DPI as the focus because it is the time point at which the dual-graph design provides the largest accuracy gain (Section 3.2) and at which both core (ISD3c) and penumbral (ISD3p) compartments are present. Cluster-to-domain purity was high (median 0.92), indicating that the unsupervised clusters closely correspond to expert-annotated domains. We emphasize that, because the differentially expressed genes and the pathway enrichment below are computed on clusters that the model itself defines, this analysis is a *descriptive* confirmation that the recovered domains carry coherent biology, a sanity check on the clusters, and is not an independent validation of accuracy over the baselines (clustering quality is quantified by ARI/NMI in Section 3.2); reported DE *p* values and enrichment FDRs should be read in that descriptive light.

Ischemic core clusters (ISD3c, purity >0.92) were characterized by cathepsin family genes (*Ctsb, Ctsd*, and *Ctsz*; lysosomal degradation), *Spp1*/osteopontin (log_2_FC = 4.75; macrophage-mediated inflammation), *Lgals1* (galectin-1; immune regulation), and collagen genes (*Col3a1, Col1a2*; fibrotic remodeling). Penumbra clusters showed complement cascade activation (*C1qa, C1qb*, and *C1qc*) alongside cathepsins and *Spp1*, consistent with active microglial phagocytosis. Glial scar domains expressed extracellular matrix genes (*Fmod, Efemp1*), *Ptgds* (prostaglandin D synthase; astrocytic), and growth factors (*Igf2, Ptn*). Lesioned cortex clusters were enriched for reactive astrocyte markers (*Serpina3n, Fabp7*) and cell-cycle genes (*Ube2c, Pbk*), indicating proliferating reactive glia. Anatomical regions retained expected marker profiles: striatum clusters expressed *Ppp1r1b* (DARPP-32), *Penk*, and *Gpr88* (medium spiny neuron markers); fiber tract clusters showed oligodendrocyte markers (*Plp1, Mog*, and *Cldn11*); and cortex clusters expressed excitatory neuron markers (*Slc17a7* and *Camk2a*).

Spatial expression patterns of representative markers across all four samples confirm domain-specific localization (Supplementary Figure S3). *Spp1* is largely absent in control tissue but is strongly induced within the ischemic territory at 1 DPI and 3 DPI, fading by 7 DPI. *C1qa* expands more diffusely from a baseline microglial pattern into a stronger lesion-associated signal in injury samples. *Serpina3n* is restricted to lesion borders and the glial scar, consistent with reactive astrogliosis. The anatomical markers *Ppp1r1b, Plp1*, and *Slc17a7* retain their expected restriction to striatum, fiber tracts, and cortex, respectively, across all four time points. Together, these signatures are consistent with the known molecular pathology of MCAO stroke progression (Dirnagl et al., 1999; Iadecola and Anrather, 2011; Shi et al., 2019).

### 3.11 Pathway enrichment of lesion-associated domains

To move beyond individual marker genes, we performed over-representation analysis (Enrichr; GO Biological Process, KEGG, and Reactome) on the up-regulated differentially expressed genes of each lesion-domain category at 3 DPI (Supplementary Figure S4). The enriched pathways recapitulate the expected biology of each compartment and add a functional layer to the marker-level results. Ischemic penumbra domains were most strongly enriched for innate immune and lysosomal programs (Reactome “Innate Immune System,” KEGG “Lysosome,” “Neutrophil degranulation;” FDR < 10^−8^), consistent with active microglial and phagocytic responses at the lesion border. Glial scar domains were enriched for vascular and blood–brain-barrier transport, coagulation regulation, and IGF receptor signaling, consistent with neurovascular remodeling and scar formation. Lesioned cortex domains were enriched for cell-cycle and mitotic processes (mitotic anaphase/metaphase, chaperonin-mediated tubulin folding), consistent with proliferating reactive glia. Ischemic core domains were dominated by protein-metabolism and translation programs. These enrichments are consistent with the established molecular pathology of MCAO progression (Dirnagl et al., 1999; Iadecola and Anrather, 2011; Shi et al., 2019) and confirm that the unsupervised domains carry coherent, compartment-specific functional signatures.

## 4 Discussion

SpatialDomainAE addresses two limitations of many spatial domain identification methods: reliance on spatial proximity graphs alone and fixed fusion of spatial and expression information.

The principal finding is that the dual-graph design is most useful when tissue architecture is strongly disrupted. Across the full four-sample MCAO time course, SpatialDomainAE is competitive on average, it leads the single-graph baselines on mean ARI and is marginally behind the dual-view Spatial-MGCN, rather than uniformly dominant, and on the intact-laminar DLPFC benchmark it performs comparably to most spatial baselines but below GraphST. The decisive distinction appears at 3 DPI, where the lesion has consolidated into core and penumbral compartments with related but separable transcriptomic programs: on this sample SpatialDomainAE significantly exceeds every external baseline, including the dual-view Spatial-MGCN (*p* = 0.037), and the controlled experiments show that the long-range transcriptomic content of the feature graph, not merely its reach, drives this gain. This pattern indicates that spatial context is not universally beneficial but becomes valuable, together with long-range transcriptomic edges, when expression and geometry alone do not cleanly separate neighboring pathological regions. The encoder and decoder are kept intentionally lightweight: higher-capacity encoders can raise absolute accuracy but tend to do so independently of the graph structure, which would obscure the dual-graph contribution that is the focus of this study.

The feature graph provides a complementary signal to local spatial proximity. In pathological tissue, physical neighbors can belong to different injury states, whereas physically distant spots may share a damage-associated transcriptional program. The long-range feature-graph edges observed in Supplementary Figure S2 are therefore not only a technical property of the graph construction; they are a mechanism by which the model can connect separated tissue regions with similar molecular states. Similar motivations underlie recent dual-graph and multi-modal fusion methods in spatial multi-omics and single-cell multi-modal analysis (Long et al., 2024; Huang et al., 2025; Meng et al., 2025; Xiao et al., 2025), but the present study focuses this idea on ischemic tissue disorganization.

The attention fusion weights add an interpretability layer that is not available in single-graph methods such as STAGATE, GraphST, and SpaGCN. We interpret α_feature_ as a model-derived summary of how much each spot relies on transcriptomic similarity rather than local tissue geometry. This readout should not be treated as a direct measurement of tissue damage, but it follows a biologically plausible pattern: penumbral regions show elevated feature-graph reliance relative to intact anatomy, and the ischemic core follows a non-monotonic trajectory consistent with acute disorganization, lesion consolidation, and partial remodeling after MCAO (Dirnagl et al., 1999; Iadecola and Anrather, 2011). The value of this readout is that it connects the model's internal fusion behavior to spatial pathology, rather than reporting clustering accuracy alone.

### 4.1 Limitations and future work

The dataset contains four samples from one MCAO protocol; validation on additional stroke models and other pathologies would strengthen generalizability. The reconstruction loss (MSE) does not explicitly account for the count nature of gene expression data; replacing it with a negative binomial or ZINB likelihood, or adding a spatial smoothness regularizer, may improve embedding quality. Incorporating histological features as a third graph modality (Xiao et al., 2025; Xu et al., 2022) and applying inductive architectures (Hamilton et al., 2017) to process new samples without retraining are directions worth pursuing. Integration with multi-modal fusion frameworks (Meng et al., 2024; Huang et al., 2025) could extend the approach to spatial multi-omics data.

### 4.2 Conclusion

SpatialDomainAE is a dual-graph attention autoencoder for unsupervised spatial domain identification in spatial transcriptomics. On a mouse MCAO ischemic stroke dataset it is competitive on average with strong external baselines, comparable to the dual-view Spatial-MGCN and ahead of STAGATE, GraphST, and SpaGCN on the four-sample mean, and significantly exceeds all of them specifically at the most disrupted time point (3 DPI), including the dual-view Spatial-MGCN. Its distinctive contribution is mechanistic and practical rather than a large average-accuracy gain: controlled experiments show that the long-range *transcriptomic* content of the feature graph, not merely its reach, drives the 3 DPI improvement, and the method is robust to a fixed, untuned clustering resolution. As an exploratory analysis the per-spot fusion weights separate lesion-associated domains at the region level; an annotation-independent control indicates they do *not* track a fine-scale disorganization measure, so they are presented as a model diagnostic rather than a validated biomarker. Differential expression and pathway enrichment support the biological coherence of the inferred domains. The advantage is specific to the disrupted-tissue regime and does not extend to intact laminar cortex, delimiting where the method is most useful.

## Data Availability

Publicly available datasets were analyzed in this study. This data can be found here: https://www.ncbi.nlm.nih.gov/geo/query/acc.cgi?acc=GSE233815 [Gene Expression Omnibus (GEO) GSE233815].
