# Supplementary material for: Dual-graph attention autoencoder for spatial domain identification in ischemic stroke
Source: Front Neurosci. 2026 Jul 31;20:1881087. doi: 10.3389/fnins.2026.1881087 (PMC13472989; doi:10.3389/fnins.2026.1881087)
Supplement: Supplementary file 1 [file Data_Sheet_1.pdf]

Supplementary Material

Dual-Graph Attention Autoencoder for Spatial Domain Identification in Ischemic Stroke

This document contains the Supplementary Methods (baseline implementation details), Supplementary Figures S1–S4 (S1: enlarged 3 DPI lesion region; S2: physical span of the spatial and feature graph edges; S3: spatial expression of representative domain markers; S4: pathway enrichment of the lesion-associated domains), and Supplementary Tables S1–S2 (S1: label-free resolution selection; S2: graph-structure ablation).

Supplementary Methods

Baseline implementation details

To make the comparison fair, every baseline received the same input as our model (the per-sample 3,000-HVG library-size-normalized, log-transformed expression matrix and the same spatial coordinates), was run over the same 10 random seeds, and had its embedding clustered with the same oracle Leiden resolution grid; ground-truth labels were used only after clustering to select the reported resolution. Method-specific settings were as follows. **STAGATE:** spatial graph from the $k_s = 15$ KNN used throughout, encoder `hidden_dims`$= [512, 30]$, 500 epochs, learning rate 10^{-3}. **GraphST:** built-in 10× Visium spatial graph, output dimension 64, 600 epochs (the input matrix was passed as pre-selected highly variable so GraphST's internal preprocessing, which expects raw counts, was bypassed). **SpaGCN:** 50 principal components, spatial adjacency computed from coordinates without histology, characteristic length l selected by its `search_l` routine ($p = 0.5$), and deep clustering with `init=kmeans` and 20 initial clusters (its native Louvain resolution search requires the `louvain` package, which is unavailable in the runtime environment). **Spatial-MGCN:** spatial graph from the $k_s = 15$ KNN and feature graph from the same expression-correlation KNN as our model, run with the authors' default training settings. **SpaMask:** run through its own pipeline (raw counts, its native 3,000-HVG selection, normalization and masking; spatial KNN with $k = 15$; 1,000 epochs), and evaluated both under the shared oracle-Leiden protocol and its native k-means clustering.

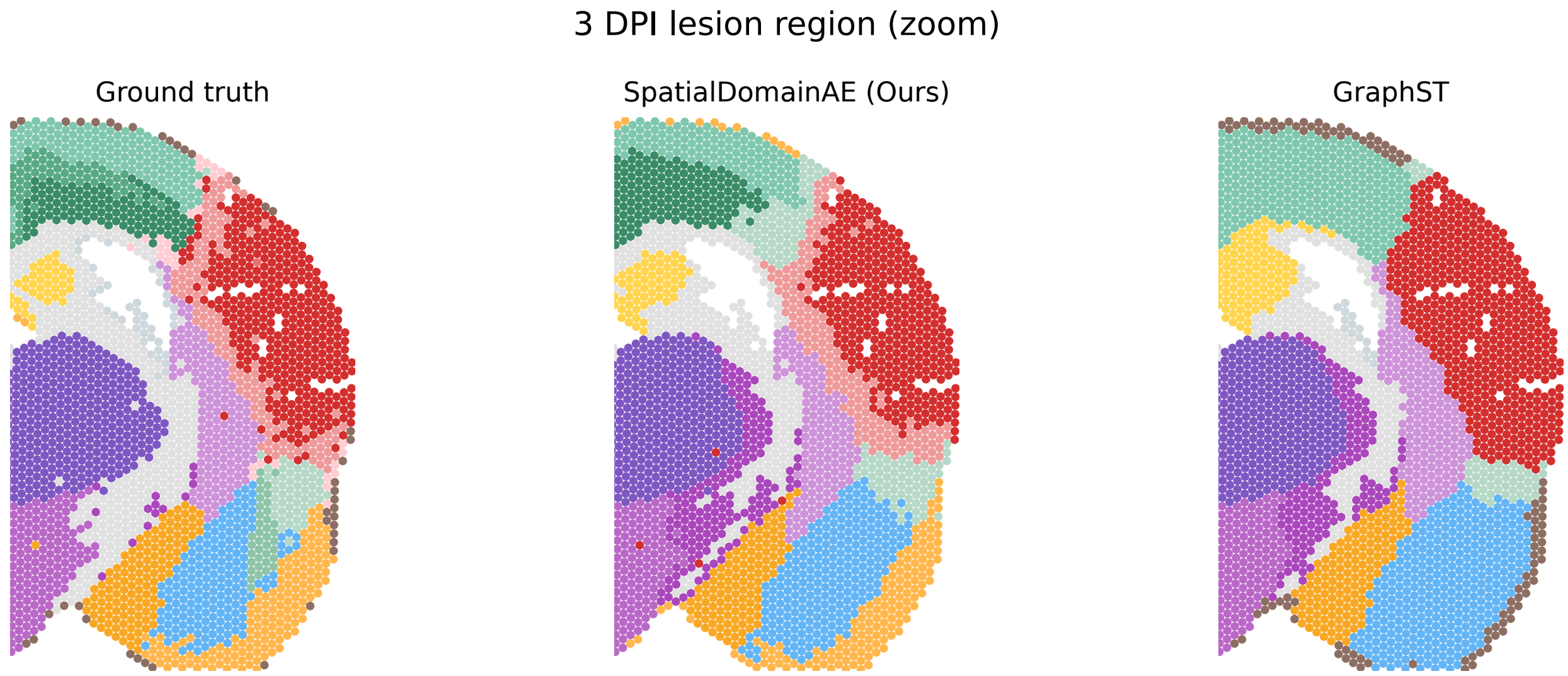

Figure S1: Enlarged view of the 3 DPI lesion-bearing region (the section with the strongest core–penumbra heterogeneity), comparing ground-truth annotations, SpatialDomainAE (Ours), and the strongest single-graph baseline (GraphST), rendered at increased spot size for clarity. SpatialDomainAE more faithfully recovers the ischemic core/penumbra (red domains) and the surrounding lesioned cortex than GraphST, which fragments the lesion territory. This figure supports Section 3.1 of the main text.

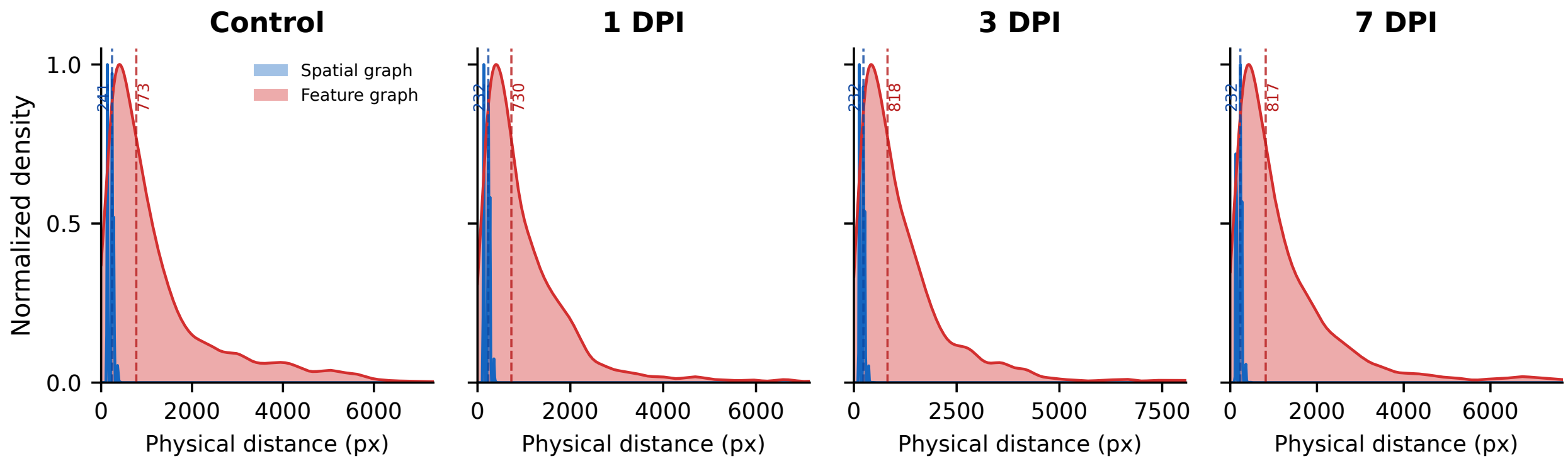

Figure S2: Physical distance distribution of graph edges, one panel per sample. Spatial graph edges (blue) cluster near the Visium spot pitch (median ∼232–241 pixels, ∼100 μm); feature graph edges (red) span ∼3.3× longer distances (median ∼730–818 pixels), capturing transcriptomic similarity between physically distant spots that no single spatial-only graph could reach. Vertical dashed lines mark per-graph medians; KDE curves are normalized to the same maximum within each panel for visibility. This figure supports Section 3.5 of the main text.

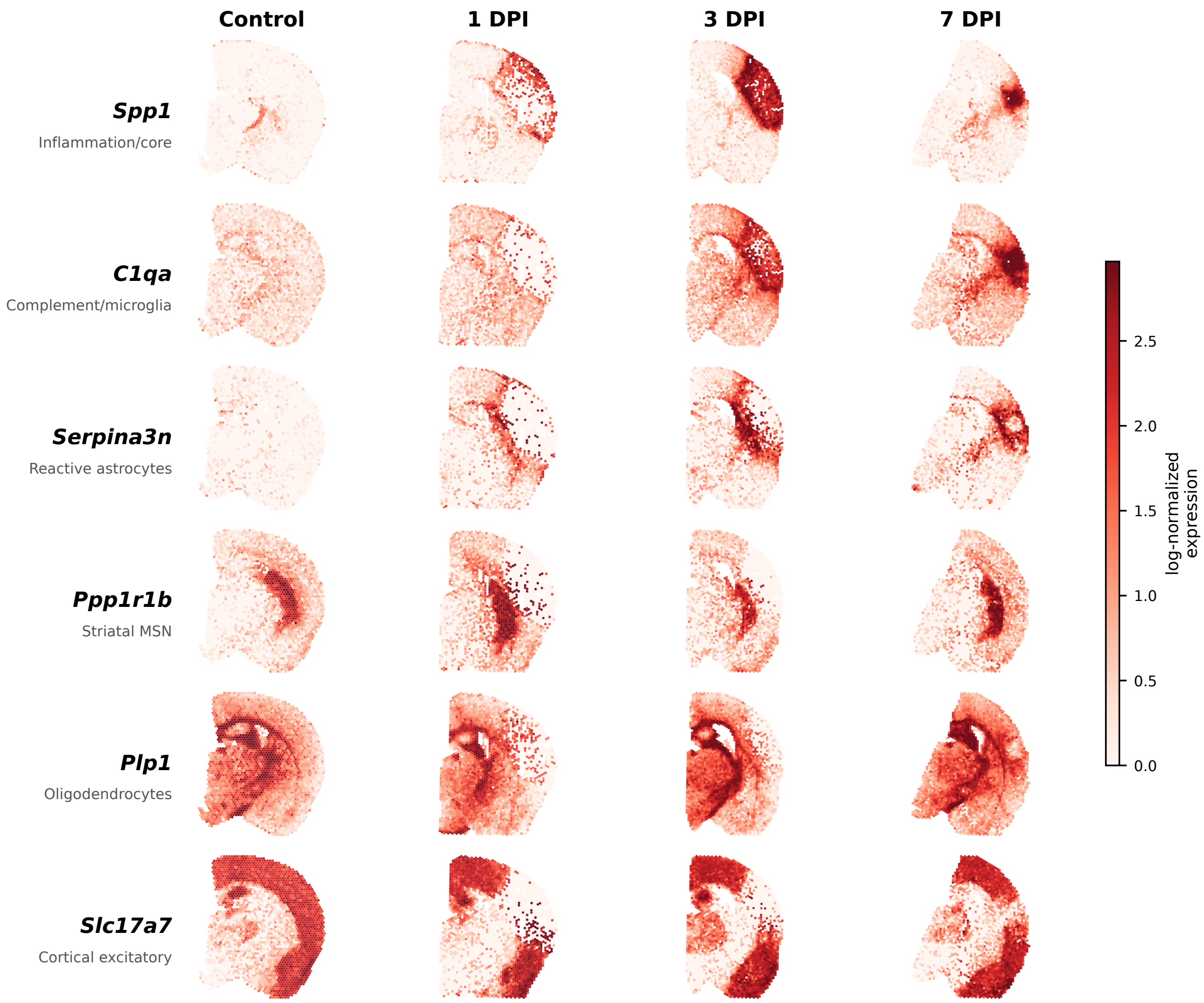

Figure S3: Spatial expression of representative domain markers across the four samples. Each row shows the log-normalized expression of one marker gene; columns are time points. Lesion-associated markers (*Spp1*, *C1qa*, *Serpina3n*) are absent or low in control tissue and become prominent within the ischemic territory after injury. Anatomical markers (*Ppp1r1b*, *Plp1*, *Slc17a7*) retain their expected localization to striatum, fiber tracts, and cortex, respectively. All panels share the same color scale clipped at the per-gene 99th percentile. This figure supports the marker-gene analysis of the main text.

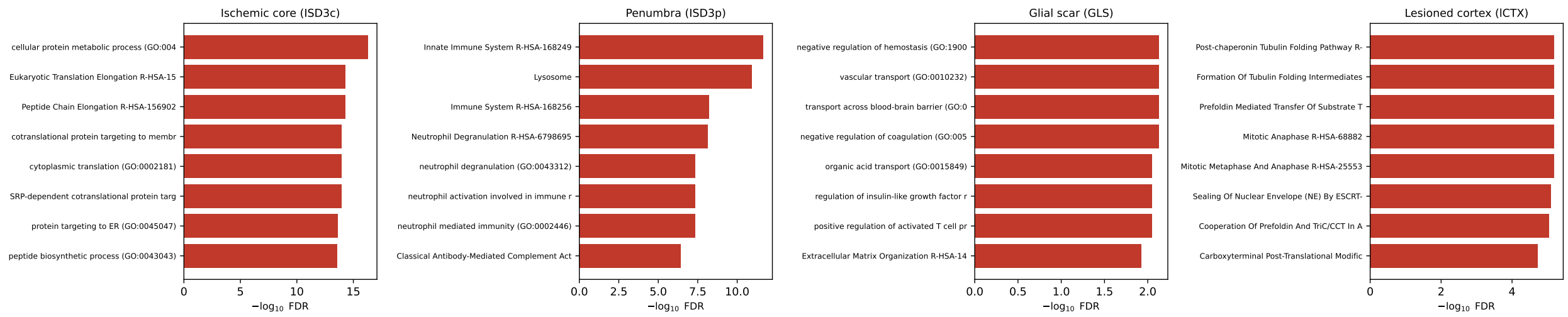

Figure S4: Pathway enrichment of up-regulated differentially expressed genes for each 3 DPI lesion-domain category (GO/KEGG/Reactome via Enrichr). Bars show the top significant terms per category ranked by $-\log_{10}$ FDR. Penumbra is dominated by innate-immune/lysosomal/neutrophil programs; glial scar by vascular and blood–brain-barrier transport; lesioned cortex by cell-cycle/mitotic processes (proliferating glia); ischemic core by protein-metabolism/translation programs. This figure supports the pathway-enrichment analysis of the main text.

Table S1: Resolution selection without oracle labels (ARI, mean over 3 seeds). *Oracle*: best-ARI resolution on the grid (uses labels; upper bound). *Fixed*: a single resolution $\gamma = 1.0$ for all methods/samples. *Silhouette*: resolution chosen by maximizing silhouette score (label-free). Best per column in **bold**. The *Oracle* columns here come from a separate three-seed run and so differ slightly from the multi-seed benchmark in Table 1 of the main text (e.g. our 3 DPI 0.680 vs. 0.700; Spatial-MGCN 0.619 vs. 0.668); within each column all methods are evaluated identically, so the column-wise comparison is valid. This table supports the resolution-selection analysis of the main text.

	3 DPI ARI			Four-sample mean ARI		
Method	Oracle	Fixed	Silh.	Oracle	Fixed	Silh.
SpatialDomainAE (Ours)	**0.680**	**0.666**	0.593	0.605	**0.585**	0.545
Spatial-MGCN	0.619	0.499	**0.613**	**0.608**	0.486	**0.605**
SpaGCN	0.617	0.524	0.606	0.600	0.508	0.593
GraphST	0.641	0.617	0.580	0.581	0.536	0.532
STAGATE	0.561	0.435	0.437	0.508	0.406	0.274

Table S2: Ablation of graph structure within our backbone (mean±SD). All variants share the same encoder, pipeline, and clustering. As an internal comparison among our own variants, this is evaluated over 20 runs (10 seeds × 2 repeats); the main benchmark (Table 1) uses 10 seeds for parity with the external baselines. Best per column in **bold**, second-best underlined. The dual graph significantly improves mean ARI over the spatial-only variant (paired Wilcoxon over 10 seeds, $p = 0.010$); the gain over no-graph is small and not significant ($p = 0.064$). The content-specific contribution of the feature graph is shown directly by the controlled experiments in main-text Table 2.

Variant	3 DPI ARI	Mean ARI	Mean NMI
SpatialDomainAE (dual graph)	**0.701±0.026**	**0.606±0.014**	**0.739±0.006**
SpatialGATAE (spatial only)	0.687±0.021	0.590±0.008	0.726±0.005
ExprOnlyAE (no graph)	0.696±0.026	0.595±0.011	0.728±0.008
